# Diagnosis of acute heart failure in CT pulmonary angiography: feasibility and accuracy

**DOI:** 10.1007/s00330-022-08676-9

**Published:** 2022-03-16

**Authors:** Ilaria Vittoria de Martini, Adrian Raoul Kobe, Christian Roeren, Robert Manka, André Euler, Dagmar I. Keller, Frank Ruschitzka, Hatem Alkadhi, Matthias Eberhard

**Affiliations:** 1 Institute of Diagnostic and Interventional Radiology, University Hospital Zurich, University of Zurich, Raemistrasse 100, 8091 Zurich, Switzerland; 2 Department of Cardiology, University Heart Center, University Hospital Zurich, University of Zurich, Zurich, Switzerland; 3 Emergency Department, University Hospital Zurich, University of Zurich, Zurich, Switzerland

**Keywords:** Computed tomography, Computed tomography angiography, Dyspnea, Pulmonary embolism, Heart failure

## Abstract

**Objectives:**

To evaluate the feasibility and accuracy of diagnosing acute heart failure (HF) with CT pulmonary angiography (CTPA) in emergency department patients.

**Methods:**

In this retrospective single-center study, we evaluated 150 emergency department patients (mean age 65 ± 17 years) undergoing CTPA with a fixed scan (100 kVp) and contrast media protocol (60 mL, 4 mL/s) who had no pulmonary embolism (PE). Patients were subdivided into training cohort (*n* = 100) and test cohort (*n* = 50). Three independent, blinded readers measured the attenuation in the right ventricle (RV) and left ventricle (LV) on axial images. The ratio (HU_ratio_) and difference (HU_diff_) between RV and LV attenuation were calculated. Diagnosis of acute HF was made on the basis of clinical, laboratory, and echocardiography data. Optimal thresholds, sensitivity, and specificity were calculated using the area under the curve (AUC) from receiver operating characteristics analysis.

**Results:**

Fifty-nine of the 150 patients (40%) were diagnosed with acute HF. Attenuation measurements showed an almost perfect interobserver agreement (intraclass correlation coefficient: 0.986, 95%CI: 0.980–0.991). NT-pro BNP exhibited moderate correlations with HU_ratio_ (*r* = 0.50, *p* < 0.001) and HU_diff_ (*r* = 0.50, *p* < 0.001). In the training cohort, HU_ratio_ (AUC: 0.89, 95%CI: 0.82–0.95) and HU_diff_ (AUC: 0.88, 95%CI: 0.81–0.95) showed a very good performance to diagnose HF. Optimal cutoff values were 1.42 for HU_ratio_ (sensitivity 93%; specificity 75%) and 113 for HU_diff_ (sensitivity 93%; specificity 73%). Applying these thresholds to the test cohort yielded a sensitivity of 89% and 89% and a specificity of 69% and 63% for HU_ratio_ and HU_diff_, respectively.

**Conclusion:**

In emergency department patients undergoing CTPA and showing no PE, both HU_ratio_ and HU_diff_ have a high sensitivity for diagnosing acute HF.

**Key Points:**

*• Heart failure is a common differential diagnosis in patients undergoing CT pulmonary angiography.*

*• In emergency department patients undergoing CT pulmonary angiography and showing no pulmonary embolism, attenuation differences of the left and right ventricle have a high sensitivity for diagnosing acute heart failure.*

## Introduction

Acute heart failure (HF) is a complex and heterogeneous syndrome being associated with high rates of morbidity and mortality, with reported 1-year mortality rates reaching 10 to 30 % [1, 2]. HF is characterized by typical symptoms and signs including dyspnea, fatigue, and peripheral edema caused by a structural and/or functional cardiac abnormality reducing cardiac output and/or increasing intracardiac pressures [3]. Acute HF is defined as new or worsening of symptoms and signs of HF representing the most frequent cause of unplanned hospital admission in patients over 65 years of age [4].

Pulmonary embolism is another life-threatening condition and the third leading cause of cardiovascular-related death [5] with an all-cause 30-day mortality rate of 5% in treated patients [6]. The diagnosis of pulmonary embolism remains challenging due to often non-specific clinical signs and symptoms, of which the most common is dyspnea. Some authors therefore suggested that pulmonary embolism should be suspected in all patients presenting to the emergency department with progressive dyspnea, chest pain, or sustained hypotension and no obvious alternate cause [7–9]. Because of the overlap of the main symptoms of HF and pulmonary embolism [8, 10], the distinction of these two entities may be hampered in the emergency setting.

Computed tomography pulmonary angiography (CTPA), owing to its high sensitivity and specificity as well as wide availability, represents the imaging modality of choice for the diagnosis or exclusion of pulmonary embolism [5, 11, 12]. The scan initiation of CTPA is usually performed with the bolus tracking technique which enables an individualized optimization of the scan start in relation to the contrast arrival in the pulmonary artery circulation [13, 14]. Still, there are numerous factors affecting the timing of contrast enhancement in CTPA which are not being taken into account entirely with bolus tracking. One of the most relevant patient-related factors is the cardiac output which determines the time of contrast arrival and the time to peak attenuation of the contrast bolus [15, 16]. For example, in patients with reduced cardiac output, the time to reach the attenuation threshold for starting the scan is delayed, similar to the time to peak attenuation which may not be covered optimally with a fixed post-trigger delay as it is standard for bolus tracking [17, 18].

The purpose of our study was to evaluate the feasibility and accuracy of diagnosing HF using attenuation measurements in the right and left ventricles (RV and LV) in patients with negative CTPA for pulmonary embolism. Our hypothesis was that a high contrast attenuation in the right heart on CTPA examinations may indicate acute HF owing to the reduced cardiac function.

## Material and methods

### Patient population

This single-center, retrospective study was approved by the local ethics committee; written consent requirement was waived. The investigation conformed with the principles outlined in the Declaration of Helsinki.

Our radiology information system was searched for patients undergoing CTPA with the identical scan and contrast media protocol in our emergency department between November 2019 and June 2020, yielding a total of 218 patients. Patients with suspicion of or with diagnosis of COVID-19 infection were excluded. Twenty-eight of the 218 patients (13%) had pulmonary embolism and were thus excluded from the study. Lack of availability of brain natriuretic peptide (NT-pro BNP) measurements on emergency department admission for the diagnosis of HF [19] was another exclusion criterion (*n* = 21, 11%). Sixteen of the remaining 169 patients (9%) had to be excluded as no echocardiography was available in the emergency department or during subsequent hospitalization despite increased NT-pro BNP values. Two patients (1%) were excluded because the region of interest (ROI) for bolus tracking was erroneously positioned in the ascending aorta, and one patient (1%) was excluded because the scan delay was manually increased by the technician.

**Fig. 1:**
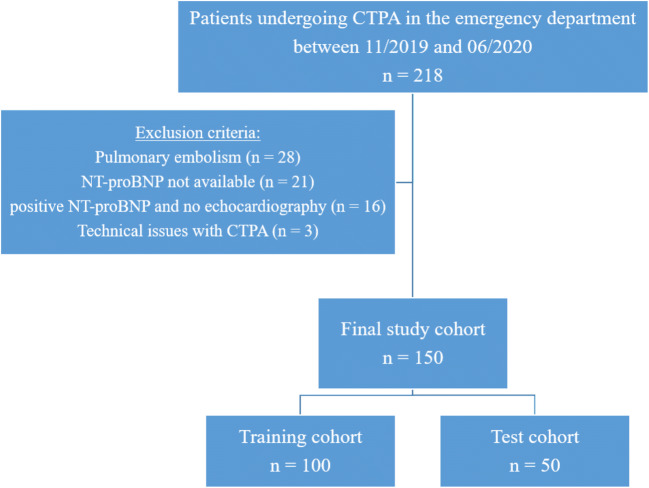
Study flow chart

Finally, a total of 150 patients (mean age 65 ± 17 years, 72 women, 78 men) were included in this study (Fig. 1). Patients were grouped according to the date of presentation, with the first 100 patients (67%) as the training cohort (scanned between November 2019 and March 2020) and the other 50 patients (33%) as the test cohort.

### CT scan protocol

Imaging was performed in the single-energy mode using a second-generation dual-source CT scanner (Somatom Definition Flash; Siemens Healthineers) with our institutional default protocol: fixed tube voltage 100 kVp, reference tube current time product 100 mAs using automatic tube current modulation (CareDose4D, Siemens Healthineers), slice acquisition 128 × 0.6 mm by means of a z-flying spot, gantry rotation time 500 ms, pitch 1, and scan direction craniocaudal.

A bolus of 60-mL iso-osmolar, nonionic iodinated contrast material (350 mg I/mL, Iobitridol, Guerbet) followed by a saline flush of 30 mL was injected into an antecubital vein with a dual-head power injector at a flow rate of 4.0 mL/s. Image initiation was controlled by bolus tracking with a ROI in the pulmonary trunk, using a 120 Hounsfield units (HU) attenuation threshold at 120 kVp. After reaching this threshold, the scan was initiated after a fixed delay of 5 s.

All images were reconstructed with sinogram affirmed iterative reconstruction (SAFIRE, Siemens Healthineers) at a strength level of 3 using a medium-smooth reconstruction kernel (I30f), a slice thickness of 2 mm, and slice increment of 1.6 mm.

### Clinical data analysis

The clinical data and electronic medical records of all 150 patients were reviewed (M.E.; 8 years of experience in cardiovascular radiology). Age, sex, hypertension, diabetes, smoking, chronic kidney disease, NT-pro BNP levels at admission, final diagnosis, and, if available, echocardiography results were noted.

### Diagnosis of HF

HF was diagnosed according to the 2016 European Society of Cardiology (ESC) guidelines [3]. According to these, assessment of HF was primarily assessed with (i) clinical history, (ii) physical examination, and (iii) electrocardiography. In patients with ≥ 1 positive item, NT-pro BNP was applied to evaluate the need for echocardiography due to its high negative predictive value [19]. The cutoff for a negative NT-proBNP was < 300 pg/mL as proposed for the acute setting according to current guidelines [3, 20]. HF with preserved ejection fraction (HFpEF) was defined as a left ventricular ejection fraction ≥ 50%, and HF with reduced ejection fraction (HFrEF) was defined as a left ventricular ejection fraction < 50%.

### Image analysis

#### Attenuation measurements

**Fig. 2:**
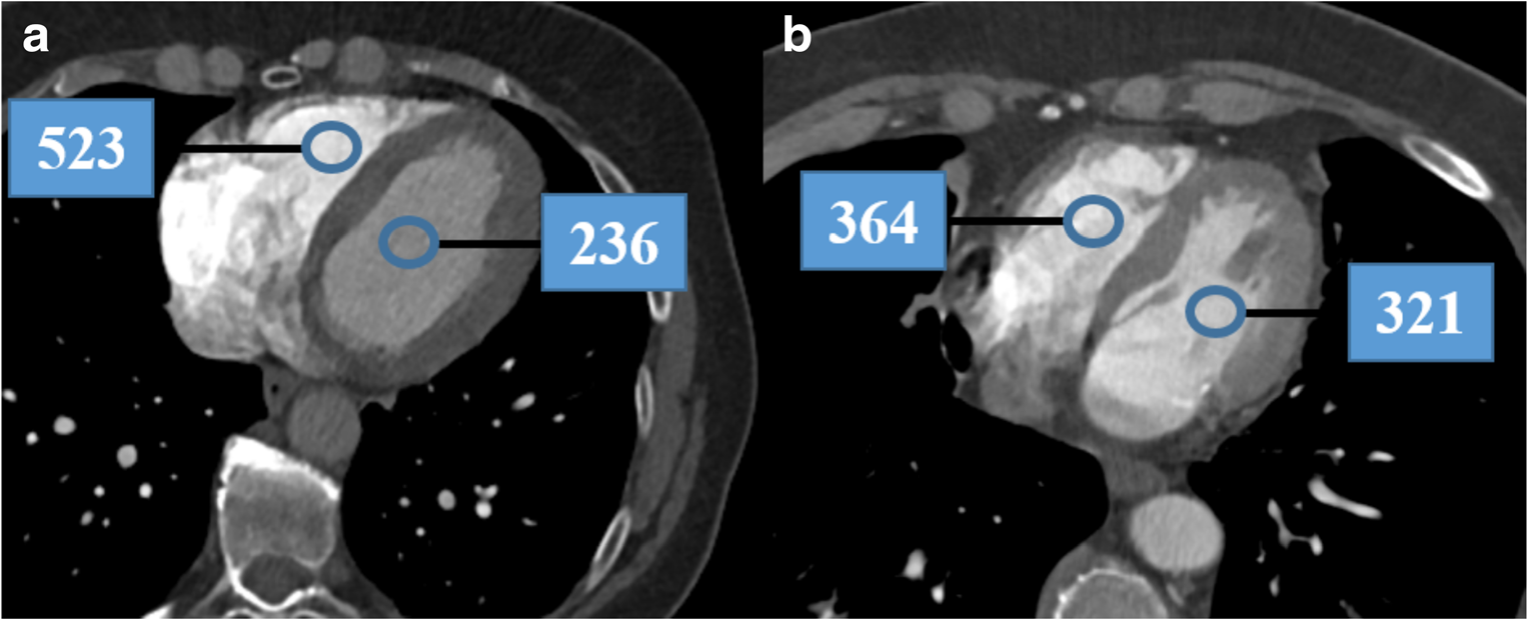
Attenuation measurements in the right and left ventricles. Panel **a** shows attenuation measurements in a 65-year-old female patient without pulmonary embolism demonstrating higher attenuation in the RV than in the LV (RV: 523 HU, LV: 236; HU_ratio_: 2.22, HU_diff_: 287). This patient was subsequently diagnosed with acute heart failure and a left ventricular ejection fraction of 48%. Panel **b** shows attenuation measurements in a 70-year-old female patient without pulmonary embolism demonstrating comparable attenuation in both ventricles (RV: 364 HU, LV: 321 HU; HU_ratio_: 1.13, HU_diff_: 43). This patient was diagnosed with acute exacerbation of chronic obstructive pulmonary disease

All CT images of the training cohort were analyzed and evaluated by two blinded and independent readers (I.V., A.K.; both with 4 years of experience in cardiovascular imaging). The two readers measured the attenuation (in HU) using a ROI (10 mm in diameter) in the RV and LV on axial CT images (Fig. 2), carefully avoiding the inclusion of the mitral or tricuspid valves, papillary muscles, the moderator band, and ventricular trabeculae. Each reader independently selected the specific axial slice for the measurements. The HU difference of the values in the RV and LV (subsequently called HU_diff_) and the ratio between the two values (RV/LV, HU_ratio_) were calculated.

An independent third reader (C.R., 1 year of experience in radiology), blinded for clinical data, performed the same measurements in the test cohort.

#### Diameter measurements

**Fig. 3:**
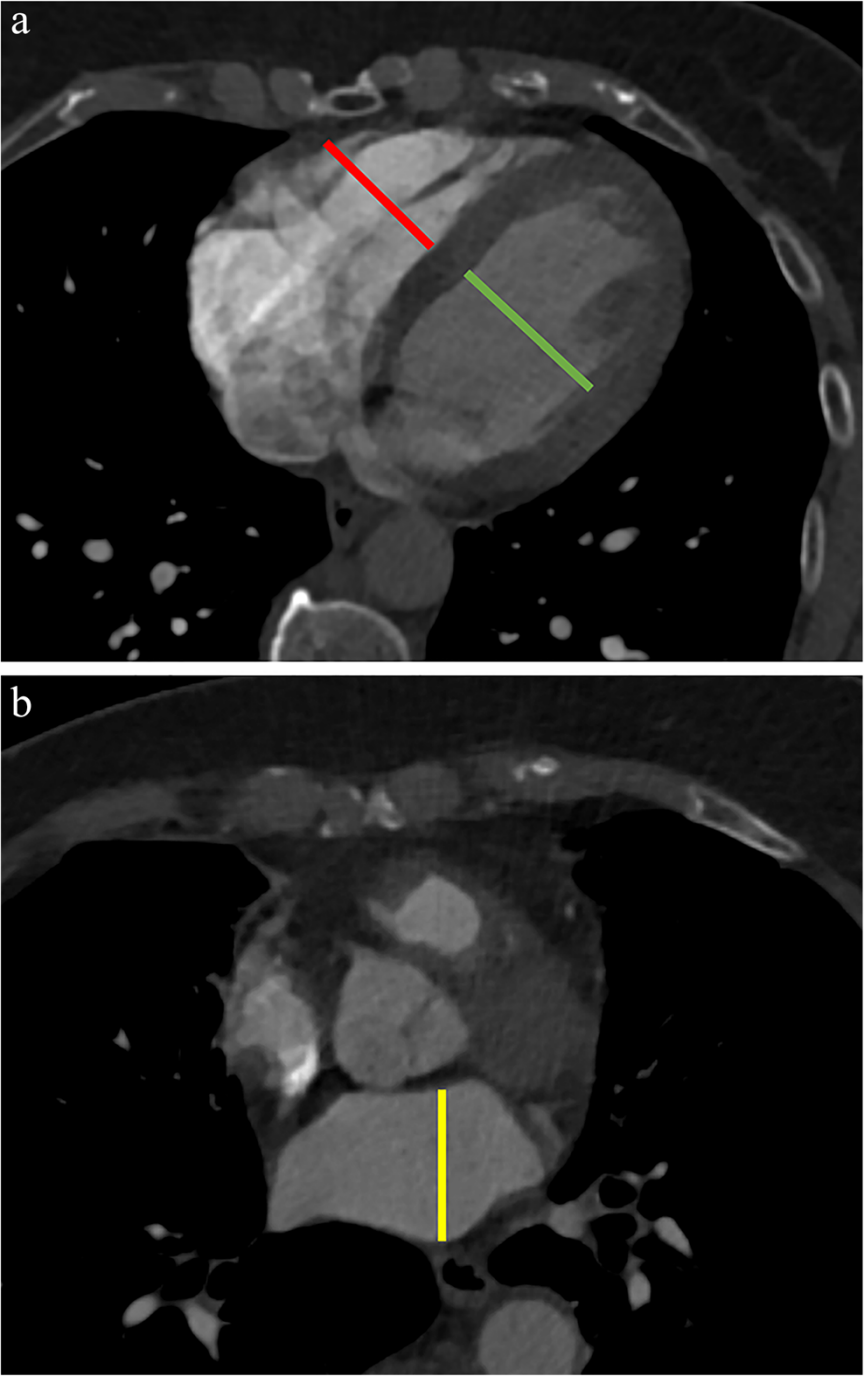
Panel **a** shows diameter measurements of the LV and RV at the widest point at the midventricular level. Panel **b** shows the LA diameter measurements at the widest antero-posterior diameter in its middle 50%

One reader (C.R., 1 year of experience in radiology) performed diameter measurements of the LV, RV, and left atrium (LA) as previously described [21, 22]. LV and RV diameters were measured at their widest point at the midventricular level, on the same CT axial image (Fig. 3A) [21]. LA size was measured using the maximum anterior-posterior diameter in its middle 50% (Fig. 3B) [22].

### Statistical analysis

Normally distributed data is given as mean ± standard deviation, non-normally distributed data is given as median [inter-quartile range], and categorical data is given as count and percentage.

Interreader agreement between the two readers in the training cohort was assessed by calculating the intraclass correlation coefficient for consistency (ICC). Parametric continuous values were compared using Student’s *t*-test or one-way analysis of variance, where appropriate. Pearson’s correlation was applied to assess correlations of continuous variables. Due to the right-skewed distribution of NT-pro BNP, correlations were calculated with log-transformed NT-pro BNP values.

The area under the curve (AUC) of receiver operating characteristics (ROC) analysis was calculated for attenuation and diameter measurements. Youden’s *J* statistic (*J* = sensitivity + specificity − 1) was applied to derive optimal thresholds to diagnose HF. Furthermore, thresholds with a high sensitivity (≥ 95%) and specificity (≥ 95%) were derived from the AUC. Using a binary logistic regression analysis, we calculated the odds ratios for the optimal thresholds of HU_diff_ and HU_ratio_ to diagnosis HF with age and sex as covariates.

A two-sided *p*-value below 0.05 was regarded as indicative of statistical significance. All analyses were performed using commercially available software (IBM SPSS Statistics for Windows, Version 25.0).

## Results

### Patient characteristics

Baseline characteristics of the entire study cohort, the training cohort, and the test cohort are presented as Table 1. On average, echocardiography was performed 1 ± 1 day after CT. HF was diagnosed in 59 of the 150 patients (40%). Thirty of the 59 patients (51%) had HFrEF, and 29 patients (49%) had HFpEF.

**Table 1:** Patient demographics. Normally distributed data is given as mean ± standard deviation, non-normally distributed data is given as median [inter-quartile range], and categorical data is given as count and percentage. Abbreviation: *BNP*, brain natriuretic peptide

	Training cohort	Test cohort	Overall
Patients	100	50	150
Females	46 (46%)	26 (52%)	72 (48%)
Age (years)	65 ± 16	65 ± 19	65 ± 17
Co-morbidities			
Hypertension	53 (53%)	28 (56%)	81 (54%)
Diabetes	26 (26%)	10 (20%)	36 (24%)
Smoker	39 (39%)	12 (24%)	51 (34%)
Chronic kidney disease	19 (19%)	11 (22%)	30 (20%)
NT-pro BNP value (ng/L)	416 [81, 1877]	237 [76, 1354]	356 [80, 1815]
Acute heart failure	41 (41%)	18 (36%)	59 (40%)
Heart failure with left ventricular ejection fraction < 50%	24 (24%)	6 (12%)	30 (20%)
Etiology of heart failure			
Ischemic	11 (11%)	6 (12%)	17 (11%)
Genetic	6 (6%)	1 (2%)	7 (5%)
Toxic	3 (3%)	2 (4%)	5 (3%)
Inflammatory	3 (3%)	0	3 (2%)
Hypertensive	7 (7%)	3 (6%)	10 (7%)
Valvular	4 (4%)	4 (8%)	8 (5%)
Arrhythmic	5 (5%)	1 (2%)	6 (4%)
Multifactorial	2 (2%)	1 (2%)	3 (2%)

### Training cohort

#### Attenuation measurements

**Fig. 4:**
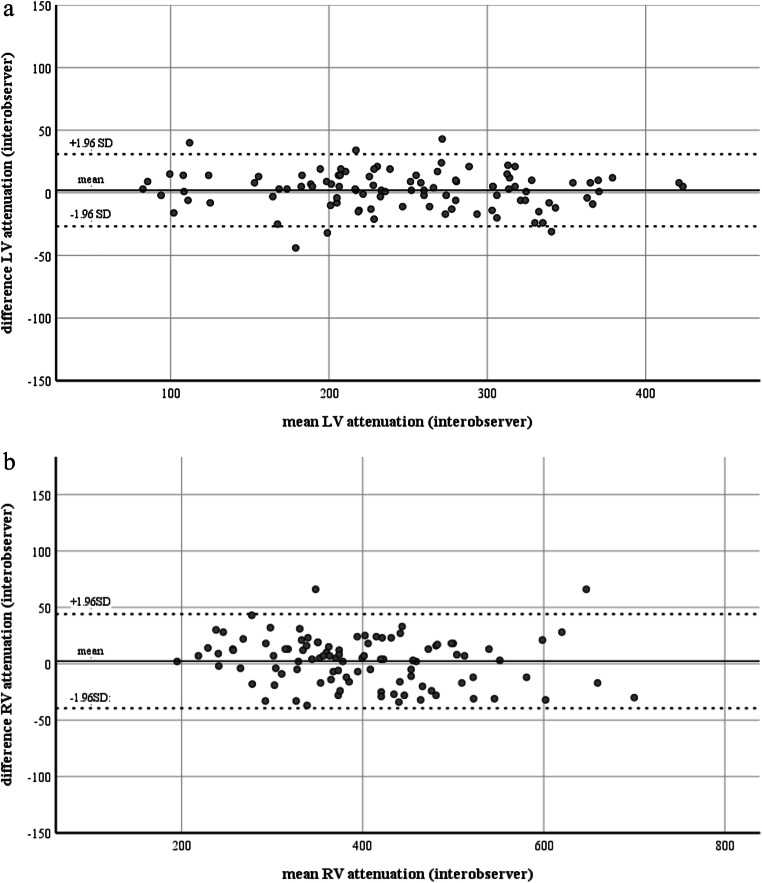
Bland-Altman plots illustrating narrow limits of agreement for attenuation measurements in the left ventricle (**a**) and right ventricle (**b**) between readers

We found an almost perfect interobserver agreement of attenuation measurements between the two readers (ICC: 0.986, 95%CI: 0.980–0.991). Bland-Altman plots showed narrow limits of interobserver agreement for LV (mean difference: 2 HU, limits of agreement: −39, 44 HU) and RV (mean difference: 2 HU, limits of agreement: −27, 31 HU), respectively (Fig. 4) attenuation measurements. Because of the high agreement, the mean of both readers was used for further analyses. Mean attenuation of the LV and RV was 246 ± 78 and 398 ± 103 HU, respectively. Mean HU_ratio_ was 1.89 ± 1.17 and mean HU_diff_ was 151 ± 132 HU.

There were no significant differences for HU_ratio_ (women: 1.71 ± 0.87, men: 2.02 ± 1.34; *p* = 0.19) and HU_diff_ (women: 139 ± 124 HU; men: 162 ± 138 HU; *p* = 0.39) between sexes. Weak though significant correlations were found between age and HU_ratio_ (*r* = 0.30, *p* = 0.002) and HU_diff_ (*r* = 0.39, *p* < 0.001) in the overall cohort; however, these findings were not significant for patients without HF (*p* > 0.05 for both). There were significant correlations between NT-pro BNP and HU_ratio_ (*r* = 0.50, *p* < 0.001) and HU_diff_ (*r* = 0.50, *p* < 0.001).

#### Diameter measurements

Mean diameters of the LV, RV, and LA were 40.5 ± 8.5 mm, 39.1 ± 7.8mm, and 35.3 ± 8.1mm, respectively.

#### HU_ratio_ and HU_diff_ in patients with/without HF

**Fig. 5:**
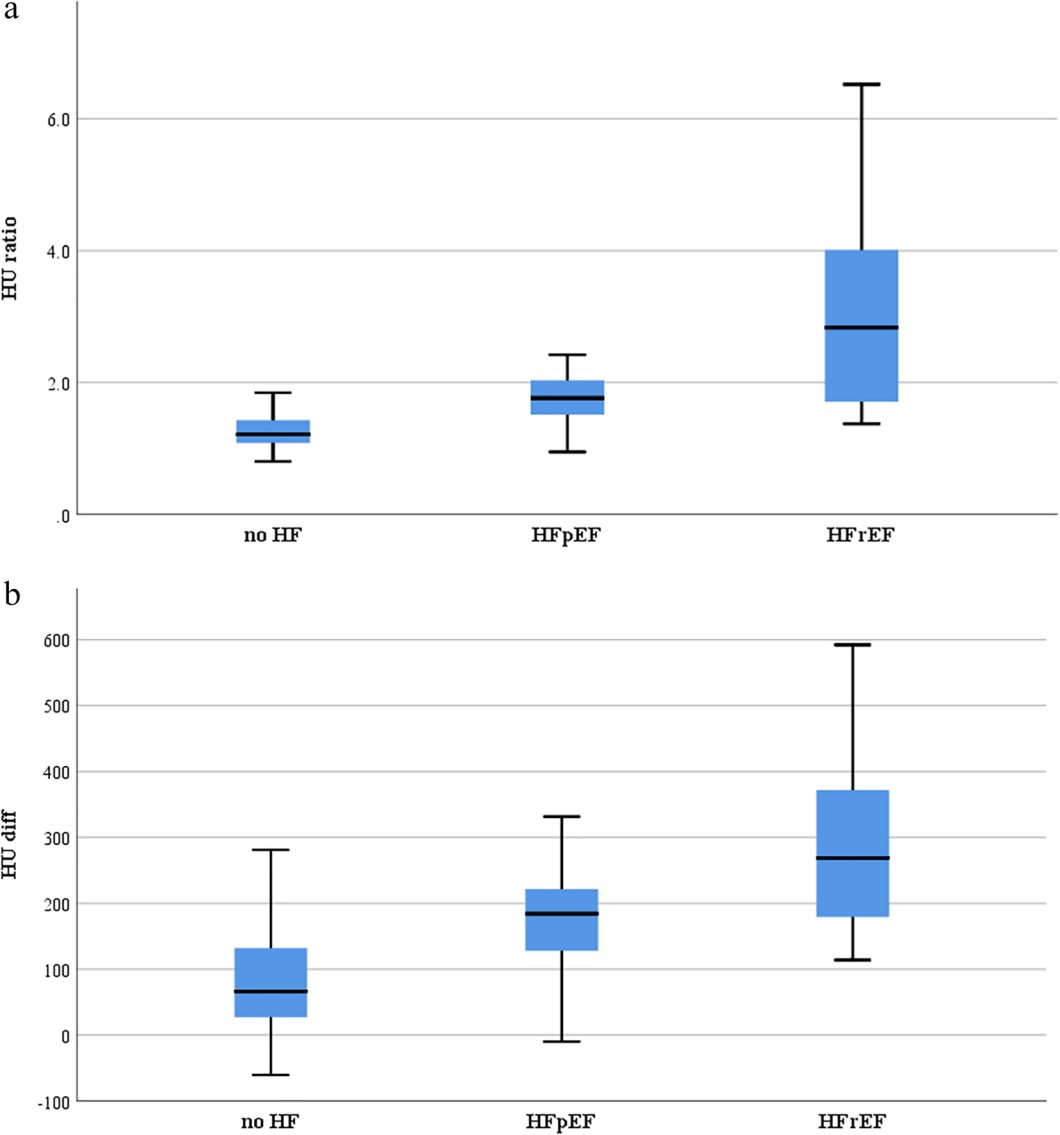
Boxplots showing the highest HF_ratio_ (**a**) and HF_diff_ (**b**) in patients with HF and reduced left ventricular ejection fraction < 50% (HFrEF; HU_ratio_: 3.19 ± 1.59, HU_diff_: 291 ± 131) compared to HF patients with preserved left ventricular ejection fraction ≥ 50% (HFpEF; HU_ratio:_ 1.99 ± 0.80, HU_diff_: 192 ± 112, *p*<0.001 and *p* = 0.006) and to patients without HF (HU_ratio_: 1.33 ± 0.34, HU_diff_: 83 ± 79, both *p* < 0.001)

HU_ratio_ (1.33 ± 0.34) and HU_diff_ (83 ± 79 HU) were significantly lower in patients without HF compared to patients with HF (HU_ratio_ 2.69 ± 1.44; HU_diff_ 250 ± 132 HU; both *p* < 0.001). Subdivision of HF patients into patients with preserved and reduced ejection fraction showed that HF_ratio_ and HF_diff_ were significantly higher in patients with HFrEF (HU_ratio_: 3.19 ± 1.59, HU_diff_: 291 ± 131) compared to HFpEF patients (HU_ratio_: 1.99 ± 0.80, HU_diff_: 192 ± 112, *p* < 0.001 and *p* = 0.006) and patients without HF (HU_ratio_: 1.33 ± 0.34, HU_diff_: 83 ± 79, both *p* < 0.001; Fig. 5).

#### Receiver operating characteristics analysis

**Fig. 6:**
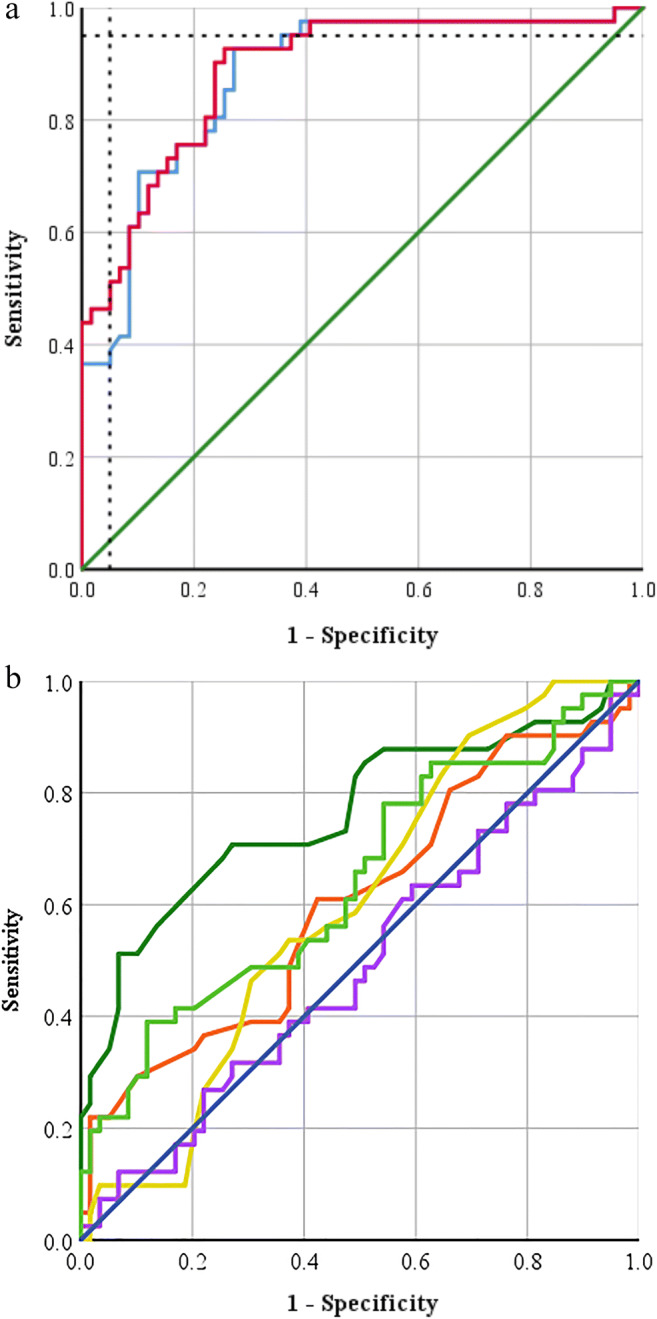
On panel **a**, receiver operating characteristics analysis illustrating the very good diagnostic performance for both HU_ratio_ (right ventricular (RV) attenuation/left ventricular (LV) attenuation; AUC: 0.89, 95% confidence interval: 0.82–0.95; red) and HU_diff_ (RV − LV attenuation; AUC, 0.88, 95% confidence interval: 0.81–0.95; blue) for diagnosing heart failure in patients undergoing CTPA and who had no pulmonary embolism. Dashed lines represent 95% specificity and 95% sensitivity. The green line represents the 45° diagonal. On panel **b**, receiver operating characteristics analysis illustrates the lower diagnostic performances of left atrial diameter (LA, dark green), left ventricular diameter (LV, orange), right ventricular diameter (RV, yellow), and LV/RV ratio (purple) and LA/RV ratio (light green) to diagnose heart failure in patients undergoing CTPA and who had no pulmonary embolism. The dark blue line represents the 45° diagonal

HU_ratio_ (AUC: 0.89, 95%CI: 0.82–0.95) and HU_diff_ (AUC: 0.88, 95%CI: 0.81–0.95) showed a very good performance to diagnose HF (Fig. 6a). In contrast, LV (AUC: 0.61, 95%CI: 0.49–0.72), RV (AUC: 0.60, 95%CI: 0.49–0.71), and LA (AUC: 76, 95%CI: 0.66–0.86) measurements as well as LA/RV (AUC: 0.64, 95%CI: 0.53–0.75) as well as LV/RV (AUC: 0.49, 95%CI: 0.37–0.61) ratios showed lower AUC values to diagnose HF (Fig. 6b).

#### Derivation of thresholds

Optimum cutoff values to detect HF were 1.42 for HU_ratio_ (specificity, 79% and sensitivity, 90%) and 113 for HU_diff_ (specificity, 79% and sensitivity, 92%). The odds ratio for a HU_ratio_ of 1.42 to detect HF was 27.8 (95%CI: 7.2–106.7). The odds ratio for a HU_diff_ of 113 was 27.3 (95%CI: 7.2–103.9) to detect HF. Cutoff values for HU_ratio_ and HU_diff_ providing ≥ 95% sensitivity and specificity are shown in Table 2.

**Table 2:** Attenuation measurement thresholds for HU_ratio_ (right ventricular (RV) attenuation/left ventricular (LV) attenuation) and HU_diff_ (RV − LV attenuation) derived in the training cohort

Training cohort			
	Threshold	Specificity (%)	Sensitivity (%)
HU_diff_ (in HU)			
Sensitivity > 95%	93	64	95
Optimum	113	73	93
Specificity > 95%	250	95	39
HU_ratio_			
Sensitivity > 95%	1.38	63	95
Optimum	1.42	75	93
Specificity > 95%	2.09	95	51

### Test cohort

In the test cohort, the mean attenuation in the LV and RV was 262 ± 71 HU and 393 ± 121 HU, respectively. The mean HU_ratio_ was 1.65 ± 0.91 and the mean HU_diff_ was 131 ± 130 HU.

Applying the thresholds established in the training cohort (1.42 for HU_ratio_ and 113 HU for HU_diff_) yielded a sensitivity of 89% and 89% and specificity of 63% and 69%, respectively, for the diagnosis of HF based on attenuation measurements in the RV and LV (Table 3). In the test cohort, the odds ratio for a HU_ratio_ of 1.42 to detect HF was 18.2 (95%CI: 3.2–103.5). The odds ratio for a HU_diff_ of 113 was 17.6 (95%CI: 3.0–102.7) to detect HF.

**Table 3:** Validation of specificity and sensitivity for different thresholds of HU_diff_ (right ventricular (RV) attenuation − left ventricular (LV) attenuation) and HU_ratio_ (RV attenuation/LV attenuation) in the test cohort

Test cohort		
Threshold	Specificity (%)	Sensitivity (%)
HU_diff_ (in HU)		
93	59	89
113	63	89
250	91	33
HU_ratio_		
1.38	63	89
1.42	69	89
2.09	94	39

## Discussion

CTPA is often used as the first-line imaging test in patients presenting to the emergency department complaining of dyspnea and chest pain, and/or with syncopal episodes [6]. However, detection rate of acute pulmonary embolism on CTPA may be as low as 2% depending on the institution [23–25]. The main reason for these low rates is the issue that symptoms indicating acute pulmonary embolism are often unspecific and that there exists a remarkable overlap to symptoms of other diseases such as acute HF [9].

From our initial cohort of 218 patients, the positivity rate of CTPA was 13%. Vice versa, 87% of our patients initially suspected to suffer from pulmonary embolism subsequently obtained an alternative diagnosis. Thus, finding an alternative etiology for the patients’ symptoms and signs on CTPA studies appears highly relevant. This is further substantiated by the fact that 40% of our patients undergoing CTPA and who had no pulmonary embolism were finally diagnosed with acute HF within 30 days of emergency department admission.

A sufficiently high contrast opacification of the pulmonary arteries is a prerequisite of CTPA to allow for a safe diagnosis or exclusion of pulmonary embolism [12, 25]. To reach an adequate contrast in the pulmonary arteries, several factors, including the contrast media administration protocol and the CT scanning technique as well as patient-related factors, have to be taken into account [15]. One critical patient-related factor is cardiac output, which determines the delay between starting the contrast media injection and reaching the attenuation threshold using the bolus tracking technique for timing the scan start [15]. Cardiac output also determines the time to peak attenuation, and in patients with a reduced cardiac output, the peak arterial enhancement is delayed [15, 26]. This peak enhancement can be missed when a fixed post-trigger delay is used, which is the standard in current bolus tracking algorithms [17]. Beyond influencing contrast media arrival and time to peak enhancement in the pulmonary arteries, right and left heart functions also influence the pulmonary artery to ascending aorta transit time [27]. Using time-resolved MR angiography, Lakoma et al showed that patients after Ross procedure had prolonged pulmonary transit times, compared to normal patients [27]. In this study, the pulmonary transit time showed significant correlations with left and right ventricular ejection fraction [27]. Colin et al showed that the pulmonary transit time, measured as the time interval between peak attenuation of RV and LV, was longer in HFrEF patients with pulmonary hypertension compared to HFrEF patients without pulmonary hypertension and compared to control patients [28]. Here, the pulmonary transit time also showed a significant inverse correlation with left ventricular ejection fraction and the cardiac output [28].

In line with these results, we could show significantly higher values for the difference and ratio of RV and LV attenuation in patients with both reduced systolic left ventricular function (HFrEF) and in those with preserved left ventricular function (HFpEF), as opposed to patients showing normal cardiac function. Both these parameters showed a very good diagnostic performance to diagnose HF (AUC of 0.88 and 0.89, respectively) on CTPA. The sensitivity of both parameters when using optimal thresholds was high (89–92%) and highly reproducible in different patient (training and test) cohorts. Furthermore, both difference and ratio of RV and LV attenuation showed a moderate correlation with NT-pro BNP levels supporting the value of both parameters in identifying patients with acute HF. In comparison diameter measurements of the LV, RV, and LA as well as ratios of diameter measurements showed lower diagnostic performances to diagnose HF. For these parameters, LA diameter had the highest AUC, which is compatible with previous studies [22].

Importantly, measurements of the RV and LV attenuation on axial CT images with calculation of either the ratio or difference in attenuation represents a fast and simple approach for the assessment of HF in patients undergoing CTPA, which is supported by the almost perfect interobserver agreement between readers. Thus, we introduce an easy-to-apply technique for suggesting HF in emergency department patients negative for PE in CTPA with a very good sensitivity. Introducing this technique to the emergency work-up may help accelerating the early detection and treatment of HF with the aim to resolve the patients’ symptoms.

The following study limitations must be acknowledged. First, this retrospective study included only CTPA studies performed in the emergency department of a single institution and with a limited sample size. Furthermore, we did not perform a sample size calculation for our study. Future studies with larger patient cohorts (including also a higher proportion of patients with HFrEF and HFpEF) are required to further validate the difference and ratio of attenuation in the RV and LV in such patients, which holds particularly true for the thresholds reported herein. Second, hemodynamic parameters and CT were not recorded simultaneously. Although time intervals between CT and echocardiography were short, this might have influenced our results. Third, while the CT scan and contrast media protocol were kept constant, issues regarding the contrast media application such as a potential obstruction of the venous access route with subsequent lowering of contrast media flow rates, differences in breath-holding, or contrast media extravasation may have influenced the attenuation. Furthermore, the actual numbers of LV and RV attenuation are most probably influenced by the contrast media and/or CT scanning protocol. Whether our approach is also applicable to different techniques with a more individualized scan initiation such as the test-bolus method or using a patient-specific post-trigger delay [17] must be the scope of future research. Fourth, the prevalence of HF in our patient cohort was 40% and varied between training and test cohorts. Certainly, prevalence may be different in other institutions and when applying a different patient selection.

## Conclusion

In patients undergoing CTPA in whom CT shows no pulmonary embolism, the difference and ratio of RV and LV attenuation represent a fast and simple approach to suggest acute HF as a differential diagnosis with a very good diagnostic performance. The excellent sensitivity of the two parameters when using optimal thresholds may allow for the early diagnosis of acute HF and for a prompt initiation of appropriate therapy of this highly prevalent and life-threatening disease.

## Abbreviations

AUC: Area under the curve
BMI: Body mass index
BNP: Brain natriuretic peptide
CT: Computed tomography
CTPA: Computed tomography pulmonary angiography
EF: Ejection fraction
HF: Heart failure
HU: Hounsfield unit
ICC: Intra-class correlation coefficients
LV: Left ventricle
MR: Magnetic resonance
ROC: Receiver operating characteristics
RV: Right ventricle

## Declarations

### Guarantor

The scientific guarantor of this publication is Hatem Alkadhi.

### Statistics and biometry

No complex statistical methods were necessary for this paper.

### Methodology

• retrospective

• observational

• performed at one institution

## References

[1] Arrigo M, Jessup M, Mullens W (2020). Acute heart failure. Nat Rev Dis Primers.

[2] Follath F, Yilmaz MB, Delgado JF (2011). Clinical presentation, management and outcomes in the Acute Heart Failure Global Survey of Standard Treatment (ALARM-HF). Intensive Care Med.

[3] Ponikowski P, Voors AA, Anker SD (2016). 2016 ESC Guidelines for the diagnosis and treatment of acute and chronic heart failure: the Task Force for the Diagnosis and Treatment of Acute and Chronic Heart Failure of the European Society of Cardiology (ESC) developed with the special contribution of the Heart Failure Association (HFA) of the ESC. Eur Heart J.

[4] Mebazaa A, Yilmaz MB, Levy P (2015). Recommendations on pre-hospital and early hospital management of acute heart failure: a consensus paper from the Heart Failure Association of the European Society of Cardiology, the European Society of Emergency Medicine and the Society of Academic Emergency Medicine--short version. Eur Heart J.

[5] Moore AJE, Wachsmann J, Chamarthy MR, Panjikaran L, Tanabe Y, Rajiah P (2018). Imaging of acute pulmonary embolism: an update. Cardiovasc Diagn Ther.

[6] Pollack CV, Schreiber D, Goldhaber SZ (2011). Clinical characteristics, management, and outcomes of patients diagnosed with acute pulmonary embolism in the emergency department: initial report of EMPEROR (Multicenter Emergency Medicine Pulmonary Embolism in the Real World Registry). J Am Coll Cardiol.

[7] Wells PS, Anderson DR, Rodger M (2001). Excluding pulmonary embolism at the bedside without diagnostic imaging: management of patients with suspected pulmonary embolism presenting to the emergency department by using a simple clinical model and d-dimer. Ann Intern Med.

[8] Moore C, McNamara K, Liu R (2018). Challenges and changes to the management of pulmonary embolism in the emergency department. Clin Chest Med.

[9] Agnelli G, Becattini C (2010). Acute pulmonary embolism. N Engl J Med.

[10] Rich MW (1999). Heart failure. Cardiol Clin.

[11] Esposito A, Palmisano A, Toselli M (2021). Chest CT-derived pulmonary artery enlargement at the admission predicts overall survival in COVID-19 patients: insight from 1461 consecutive patients in Italy. Eur Radiol.

[12] Monti CB, Zanardo M, Cozzi A (2021). Dual-energy CT performance in acute pulmonary embolism: a meta-analysis. Eur Radiol.

[13] Chaturvedi A, Thompson JP, Kaproth-Joslin K (2017). Identification of left ventricle failure on pulmonary artery CTA: diagnostic significance of decreased aortic & left ventricle enhancement. Emerg Radiol.

[14] Kosmala A, Gruschwitz P, Veldhoen S (2020). Dual-energy CT angiography in suspected pulmonary embolism: influence of injection protocols on image quality and perfused blood volume. Int J Cardiovasc Imaging.

[15] Bae KT (2010). Intravenous contrast medium administration and scan timing at CT: considerations and approaches. Radiology.

[16] Kovacs A, Molnar AA, Kolossvary M (2018). Genetically determined pattern of left ventricular function in normal and hypertensive hearts. J Clin Hypertens (Greenwich).

[17] Hinzpeter R, Eberhard M, Gutjahr R (2019). CT angiography of the aorta: contrast timing by using a fixed versus a patient-specific trigger delay. Radiology.

[18] Aziz W, Claridge S, Ntalas I (2019). Emerging role of cardiac computed tomography in heart failure. ESC Heart Fail.

[19] Hobbs FD, Davis RC, Roalfe AK, Hare R, Davies MK, Kenkre JE (2002) Reliability of N-terminal pro-brain natriuretic peptide assay in diagnosis of heart failure: cohort study in representative and high risk community populations. BMJ 324:1498 10.1136/bmj.324.7352.1498 PMC116449 12077039

[20] Roberts E, Ludman AJ, Dworzynski K et al (2015) The diagnostic accuracy of the natriuretic peptides in heart failure: systematic review and diagnostic meta-analysis in the acute care setting. BMJ 350:h910 10.1136/bmj.h910 PMC4353288 25740799

[21] Bax S, Jacob J, Ahmed R (2020). Right ventricular to left ventricular ratio at CT pulmonary angiogram predicts mortality in interstitial lung disease. Chest.

[22] Lick AN, Danrad R, Smith DL, Lammi MR (2017). Left atrium measurements via computed tomography pulmonary angiogram as a predictor of diastolic dysfunction. J Comput Assist Tomogr.

[23] Kline JA, Garrett JS, Sarmiento EJ, Strachan CC, Courtney DM (2020). Over-testing for suspected pulmonary embolism in American emergency departments: the continuing epidemic. Circ Cardiovasc Qual Outcomes.

[24] Penaloza A, Kline J, Verschuren F (2012). European and American suspected and confirmed pulmonary embolism populations: comparison and analysis. J Thromb Haemost.

[25] Nagel SN, Steffen IG, Schwartz S, Hamm B, Elgeti T (2019). Age-dependent diagnostic accuracy of clinical scoring systems and D-dimer levels in the diagnosis of pulmonary embolism with computed tomography pulmonary angiography (CTPA). Eur Radiol.

[26] Davarpanah AH, Hodnett PA, Farrelly CT (2011). MDCT bolus tracking data as an adjunct for predicting the diagnosis of pulmonary hypertension and concomitant right-heart failure. AJR Am J Roentgenol.

[27] Lakoma A, Tuite D, Sheehan J, Weale P, Carr JC (2010). Measurement of pulmonary circulation parameters using time-resolved MR angiography in patients after Ross procedure. AJR Am J Roentgenol.

[28] Colin GC, Pouleur AC, Gerber BL (2020). Pulmonary hypertension detection by computed tomography pulmonary transit time in heart failure with reduced ejection fraction. Eur Heart J Cardiovasc Imaging.

